# A cognitive-behavioral treatment for suicide prevention among adults with schizophrenia spectrum disorders in community mental health: Study protocol for a pilot feasibility and acceptability randomized clinical trial

**DOI:** 10.1186/s40814-024-01523-2

**Published:** 2024-07-12

**Authors:** Lindsay A. Bornheimer, Juliann Li Verdugo, Nicholas M. Brdar, Vitalis Im, Nakea Jeffers, Courtney B. Bushnell, Katie Hoener, Melisa Tasker, Krista DeWeese, Timothy Florence, Jennifer M. Jester, Cheryl A. King, Stephan F. Taylor, Joseph A. Himle

**Affiliations:** 1 School of Social Work, University of Michigan, Ann Arbor, MI USA; 2 Department of Psychiatry, Michigan Medicine, Ann Arbor, MI USA; 3 Washtenaw County Community Mental Health, Ann Arbor, MI USA

**Keywords:** Suicide, Schizophrenia spectrum disorders, Psychosis, Cognitive-behavioral therapy, Pilot clinical trial

## Abstract

**Background:**

Suicide is among the leading causes of death for adults with schizophrenia spectrum disorders (SSDs), and there is a paucity of evidence-based suicide prevention-focused interventions tailored for this vulnerable population. Cognitive-Behavioral Suicide Prevention for psychosis (CBSPp) is a promising intervention developed in the UK that required modifications for delivery in community mental health (CMH) settings in the United States of American. This pilot trial evaluates the feasibility, acceptability, and preliminary effectiveness of our modified CBSPp intervention in comparison to services as usual (SAU) within a CMH setting in a Midwestern state of the USA.

**Methods:**

This is a single-site randomized pilot trial with a planned enrollment of 60 adults meeting criteria for both SSD and SI/A. Eligible participants will be randomized 1:1 to either 10 sessions of CBSPp or SAU. Clinical and cognitive assessments will be conducted within a 4-waive design at baseline (prior to randomization and treatment) and approximately 1 month (mid-treatment), 3 months (post-treatment), and 5 months (follow-up) after baseline assessment. Qualitative interviews will also be conducted at post-treatment. The primary objective is to determine whether CBSPp is feasible and acceptable, involving examinations of recruitment rate, treatment engagement and adherence, retention and completion rates, and experiences in the CBSPp treatment and overall study. The secondary objective is to preliminarily evaluate whether modified CBSPp is associated with reductions in clinical (suicide ideation, suicide attempt, symptoms of psychosis, depression, and emergency/hospital service, hopelessness, defeat, and entrapment) and cognitive (information processing biases, appraisals, and schemas) outcomes in comparison to SAU from baseline to post-treatment assessment.

**Discussion:**

This randomized pilot trial will provide clinically relevant information about whether CBSPp can improve SI/A, depression, and psychosis among adults with SSDs. Testing this modified cognitive-behavioral suicide prevention-focused intervention has the potential for a large public health impact by increasing the intervention’s utility and usability in CMH where many individuals with SSDs receive care, and ultimately working towards reductions in premature suicide death.

**Trial registration:**

ClinicalTrials.gov NCT#05345184. Registered on April 12, 2022.

## Introduction

Suicide is a leading cause of death globally, accounting for over 703,000 deaths each year [1]. Various studies indicate that 45–90% of individuals who die by suicide have a diagnosable psychiatric illness at the time of their death [2, 3]. Of illnesses, those diagnosed with schizophrenia spectrum disorders (SSDs) have significantly greater risk for suicide as compared to those without an SSD and also the general population [4–6]. Results of a recent meta-analysis show people with SSDs and suicide thoughts were 6 times more likely to die by suicide, as compared to people with depression and suicide thoughts being 1.5 times more likely to die by suicide [7].

Suicide is the largest contributor to heightened mortality in the SSD population as compared to the general population [3], with data showing 30–60% of individuals with SSDs experience suicide thoughts, 25–50% make a suicide attempt, and approximately 5% die by suicide, all in a lifetime [8–13]. Given the high rates of suicide within SSD populations, researchers have increasingly focused on strengthening understandings of risk and protective factors, with understandings being an essential foundational step towards developing, examining, and implementing effective suicide prevention strategies [4]. Literature suggests that hopelessness, depression, age, gender, substance use, previous suicide attempt, clinical insight, substance misuse, lethality of means (e.g., firearms), and longer duration of untreated psychosis contribute to suicide risk in this population [9, 14–20].

Less has been known, however, about the ways in which positive symptoms (e.g., hallucinations and delusions) and negative symptoms (e.g., affective flattening and anhedonia) of psychosis relate to suicide risk and outcomes, with various studies demonstrating mixed findings [12, 21–27]. General population-focused suicide research and much of the serious mental illness (SMI; e.g., major depressive disorder, bipolar disorder, schizophrenia, schizoaffective disorder) literature have historically focused on depression and hopelessness as leading risk factors with less consideration of non-affective psychosis symptomatology (i.e., positive and negative symptoms) and ecologically based environmental factors. Our investigative team has contributed to providing empirical support of the relationships between positive symptoms of psychosis and increased suicide thoughts and behavior in the SSD population [5, 28–31].

Despite growing support of psychosis symptomatology’s impact on suicide thoughts and behavior [4, 10, 19, 32], there is a paucity of effective evidence-informed interventions to reduce suicide risk with considerations for psychosis symptoms. Many suicide-focused studies exclude participants with active psychosis [33], and many psychosis-focused studies exclude participants with moderate to high levels of suicide risk (e.g., Kane et al. [34, 35]; Stroup et al. [36]). Of the empirically supported interventions that target psychosis symptoms and suicide risk individually, studies on cognitive-behavioral therapies are most prevalent [4]. A robust literature supports the use of cognitive-behavioral therapy in the treatment of psychosis symptoms [37, 38], with efficacy in the reduction of both positive and negative symptoms. Similarly, a strong evidence base suggests the efficacy of cognitive-behavioral therapy for suicide prevention in decreasing experiences of suicide thoughts and behaviors [39]. However, cognitive-behavioral therapy for psychosis (CBTp) lacks an evidence base that demonstrates efficacy in reducing suicide thoughts and behaviors [4, 40, 41], and cognitive-behavioral therapy for suicide prevention (CBT-SP) does not target psychosis symptoms nor demonstrate efficacy in reducing symptoms of psychosis.

Cognitive-Behavioral Suicide Prevention for psychosis (CBSPp) was more recently developed in the UK and is one of few psychosocial interventions tailored for psychosis symptoms and aiming to prevent suicide [42–44]. Preliminary data demonstrate that CBSPp relates to mental health improvements [45–47], yet the treatment manual and provider training required modifications for an outpatient community mental health (CMH) setting in the USA where the majority of individuals with psychosis receive care and evidence-informed interventions are challenging to implement [48]. Data of prior CBSPp studies and a focus group of CMH providers highlight three important factors that contributed to the pursuit of funding to modify and test CBSPp. First, approximately 50% of CBSPp sessions were attended in prior studies with an average of 10.75 completed sessions out of the original treatment’s 24 sessions [45–47]. Second, data show that often clients do not complete CBT homework and between-session engagement can both provide a supportive reminder and positively impact clinical outcomes [42]. Accordingly, CBSPp adapted for the USA public mental health context in a prior phase of the current study [42] with promising preliminary findings in an open pilot trial [43] and in preparation for the randomized clinical trial of focus in the current protocol manuscript. Greater detail of the CBSPp treatment is presented in the methods section below.

This study aims to evaluate the feasibility, acceptability, and preliminary effectiveness of modified CBSPp in comparison to services as usual (SAU). The primary objective of this study is to examine the feasibility and acceptability of modified CBSPp. The secondary objective of this study is to preliminarily evaluate whether modified CBSPp is associated with reductions in clinical (suicide ideation and/or attempt (SI/A), symptoms of psychosis, depression, and emergency/hospital service, hopelessness, defeat, and entrapment) and cognitive (information processing biases, appraisals, and schemas) outcomes in comparison to SAU from baseline to post-treatment assessment. In addition, there will be preliminary explorations of differential response to CBSPp (moderation) and mechanisms for reducing SI/A and improving clinical and cognitive outcomes (mediation).

## Methods

**Fig. 1:**
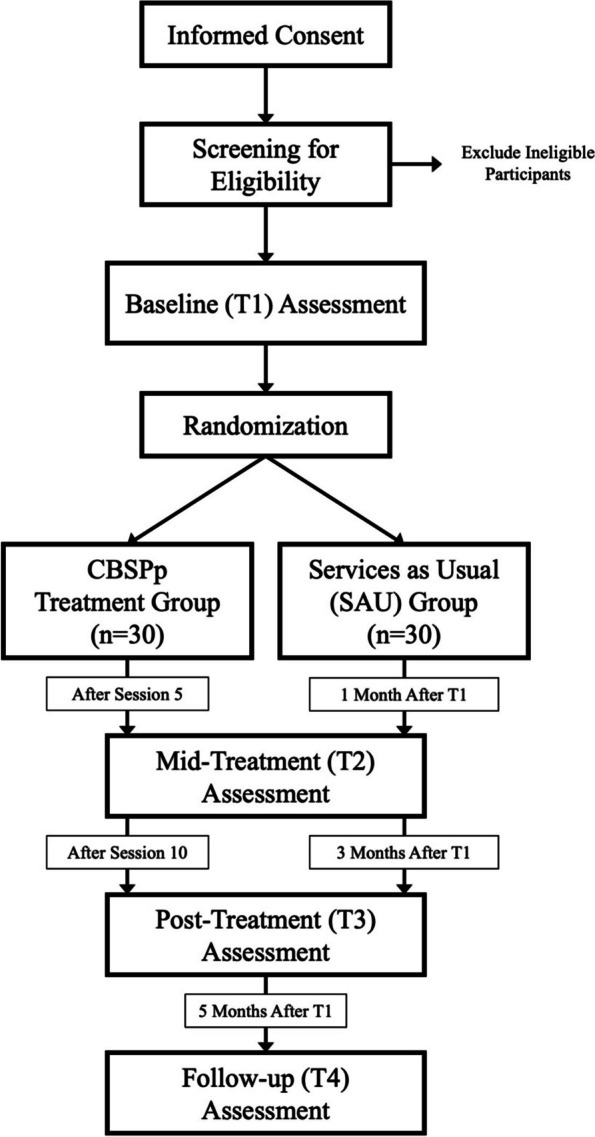
Study design flow chart

This is a pilot feasibility and acceptability randomized controlled clinical trial in which adult client participants (*n* = 60) with SSD and SI/A are randomized to either receive 10 sessions of CBSPp or SAU over a 2-year period of time. Clinical and cognitive assessments will be conducted within a 4-waive assessment design at baseline (T1: prior to randomization and treatment) and approximately 1 month (T2: mid-treatment), 3 months (T3: post-treatment), and 5 months (T4: follow-up) after baseline assessment. Qualitative interviews will be conducted at T3 for participants randomized to the CBSPp group. A study design flow chart is shown in Fig. 1.

## Study setting and sample

This single-site study is being performed by an investigative team of the University of Michigan at a Community Mental Health (CMH) setting in Michigan. This setting is the primary CMH provider in Washtenaw County and represents a public mental health system encompassing diversity across the state (e.g., race, ethnicity, socioeconomic status, organizational services, insurance/payment types). The state of Michigan has a suicide rate of 14.3 deaths per 100,000 total population, a 33% increase since 1999 [49]. This study protocol has received ethics approval from the Institutional Review Board (IRB) of the University of Michigan.

The initial client sample size (*n* = 60) was determined according to reasonable accrual and cost. Assuming 25% attrition, this will result in a final sample size of 45 at the end of the study. This meets the pilot trial sample size guidelines of Whitehead and colleagues [50] for a pilot trial with 80% power and a small effect size (0.1 ≤ δ < 0.3) for secondary clinical and cognitive outcomes, using a two-tailed alpha of 0.05. A range of 6–10 provider participants will be recruited to ensure there are available therapists for client participants when randomized to the treatment group.

Client participants will be eligible to participate if (1) over the age of 18, (2) attending and/or engaged with any services offered at the CMH setting within 6 months of screening, (3) English-speaking with at least a 6th-grade reading ability based upon the Wide Range Achievement Test 4 (WRAT-5) [51], (4) diagnosed with a schizophrenia spectrum disorder (SSD) based upon DSM-5 criteria (e.g., schizophrenia, schizoaffective, schizotypal, delusional, brief psychotic, and schizophreniform disorder) [52] using The Mini International Neuropsychiatric Interview (MINI) [53], and (5) endorsement of suicide ideation or attempt (SI/A) within 3 months of screening based upon the Columbia-Suicide Severity Rating Scale (C-SSRS) [44]. Exclusions involve the following: requiring emergency care (e.g., imminent plan with preparations for suicide attempt and intent to die) as determined by trained research staff administering the C-SSRS, or not determined to be appropriate for behavioral treatment (e.g., cognitive impairment) according to client judgment in consultation with a treating clinician at the CMH setting.

Providers delivering CBSPp will be eligible to receive CBSPp training and deliver the treatment in this study with client participants if (1) over the age of 18, (2) English-speaking, and (3) employed by the CMH setting in a mental health provider role with appropriate licensure.

## Recruitment and study procedures

Designated CMH staff will refer client participants to the research team. Clients who are interested to learn more will be instructed to either (1) reach out to the research team via phone, or (2) give consent for the CMH staff to give the client’s contact information to the research team so they can be contacted via phone and learn of next steps (eligibility, consent, enrolment, assessment). An informational conversation will be scheduled for a research team member to describe the study and review the informed consent form. Clients will be given the link to provide informed consent electronically on their own. If consent is obtained, the research team will subsequently schedule a screening assessment to determine eligibility for enrolment. Screening will involve the use of the Mini International Neuropsychiatric Interview (MINI; Sheehan, 1998) to confirm an SSD based upon criteria of the DSM-5 [52] and a 1-year history of SI/A will be confirmed using the C-SSRS [44]. Lastly, the Wide Range Achievement Test 4 (WRAT-5) [51] will be administered to confirm at least a 6th-grade reading level in English given the use of CBSPp materials. Upon completion of the T1 baseline assessments, client participants will be randomized into either the CBSPp treatment or SAU comparison group using IBM SPSS software by the project coordinator. To reduce the predictability of random sequencing, blocking using a computer-generated random number schedule will be used to determine randomization into groups.

To recruit provider participants, we will hold an informational meeting led by the principal investigator (Bornheimer) and study coordinator at the research site. Interested providers will be given information about the next steps (eligibility criteria, consent, enrollment, assessment), including a link to view and sign the electronic informed consent form. All enrolled providers will complete the CBSPp provider training and demonstrate knowledge of the intervention in exams and fidelity to the intervention in role-plays. Group supervision will be given twice a month by the principal investigator (Bornheimer). All therapy sessions will be recorded, and 20% will be randomly selected for fidelity evaluation. Providers will also complete a fidelity self-assessment at the end of each session. As indicated by fidelity evaluations, additional supervision and booster training are provided to providers individually.

## Intervention groups

Cognitive-Behavioral Suicide Prevention for psychosis (CBSPp) compared to services as usual (SAU). Importantly and for ethical reasons, participants in the CBSPp treatment group will remain in their current CMH services (e.g., medication, case management); therefore, the study groups are technically CBSPp + SAU versus SAU only (with potential to receive treatment once the clinical trial ends).

### CBSPp

CBSPp is a suicide prevention-focused individual therapy approach with specific tailoring for psychosis symptoms. The treatment was originally developed in the UK and is one of few suicide interventions tailored for adults experiencing psychosis [42, 43]. The approach uses cognitive and behavioral techniques to identify and modify suicide-related information processing biases, appraisals, and schemas [46]. CBSPp studies to date have shown preliminary improvements in global functioning and reductions in depression, hopelessness, positive and negative symptoms, suicide ideation, and suicide attempt [45–47].

Our collaborative investigative and research team modified CBSPp in a prior phase of this study [42] to adapt the approach to the USA public mental health context. Modifications were specifically made for future implementation in community mental health (CMH) settings, given the vast majority of individuals with psychosis in the USA are treated by CMH providers [48]. Community-based participatory research (CBPR) methods were used to inform CBSPp modifications, involving collaborations with stakeholders as community partners and scholarly experts in research and practice fields (see Bornheimer et al. [42], for methodological and modification details).

**Fig. 2:**
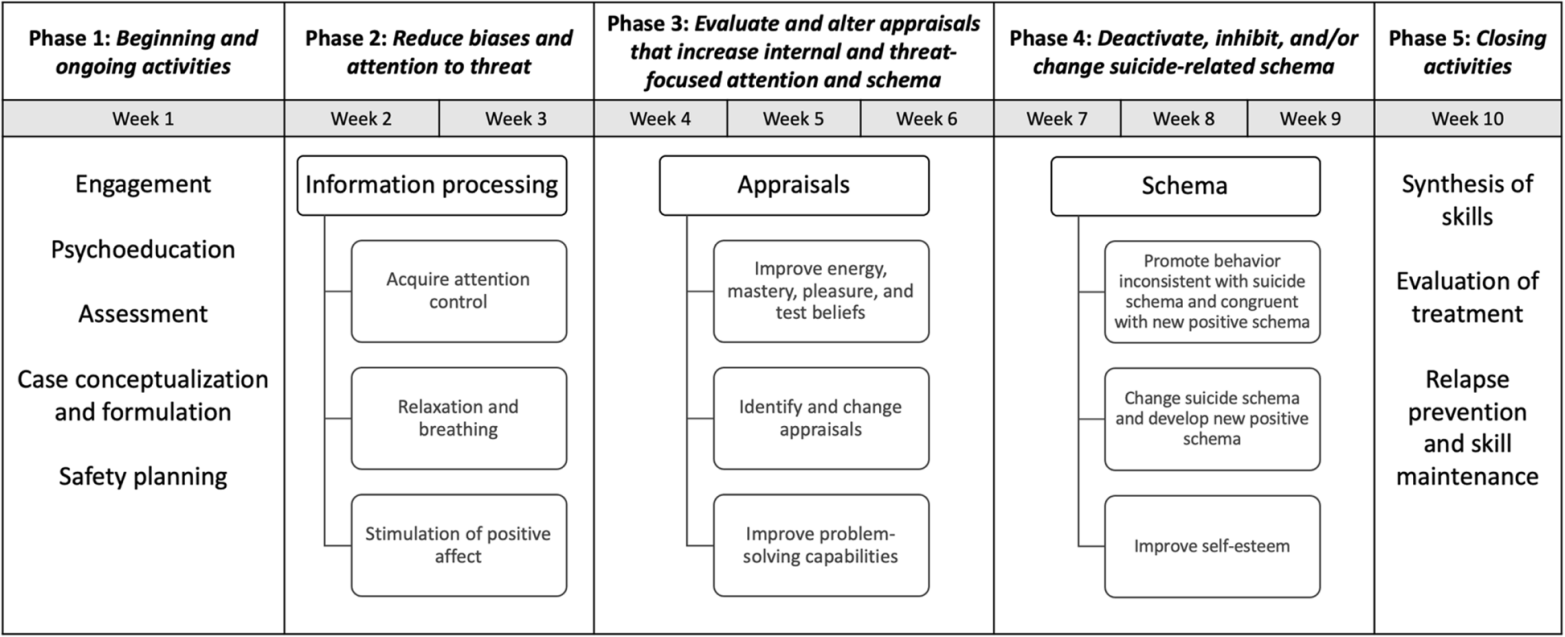
CBSPp treatment structure

The modified CBSPp consists of 10 weeks of individual therapy sessions with trained CMH provider participants. Figure 2 illustrates the structure of CBSPp by week in which there are 5 distinct phases: (1) introduction and assessment, (2) information processing, (3) appraisals, (4) schema, and (5) wrap-up and relapse prevention. Throughout the early phases of the treatment, the provider will begin to identify suicide-related information processing biases, appraisals, and schemas. Once identified, the provider will use CBSPp techniques and strategies in-session and assigned in homework to reduce suicide- and threat-focused attention, shift negative and suicide-focused appraisals, deactivate suicide schema, and adopt new beliefs and schemas of situations and the self, others, and future. Details of specific techniques and strategies are described in Tarrier et al. [46] and will be available in a future modified CBSPp treatment manual (currently in development). Exits from suicide schema will occur as evidence of resilience and the cognitive processes involved in these exits are identified and reinforced throughout treatment.

#### SAU

SAU include standard care provided by the CMH site (e.g., therapy, medication, case management, vocational services). Participants in the SAU comparison study group will continue with their current services at CMH and have the option to receive CBSPp once the clinical trial is complete. Service use data will be collected from CMH electronic medical records of all participants to allow for measurement of participant engagement in SAU.

Due to the nature of the pilot trial, client and provider participants in the study cannot be blinded for study group. Research staff scoring clinical interviews and the statistical methodologist will be blind to study group randomization.

## Assessment and measurement

Baseline assessment for clients and providers includes questions about demographic characteristics (e.g., age, gender), practice characteristics for providers (e.g., field of practice, license type, scope of work, years at CMH), and service utilization for clients (e.g., case management, medication management, therapy, and other services engaged with at CMH). A schedule of assessments and interventions for all participants is shown in Table 1. All outcomes below are assessed at T1, T2, T3, and T4 to accomplish study aims:

**Table 1:**
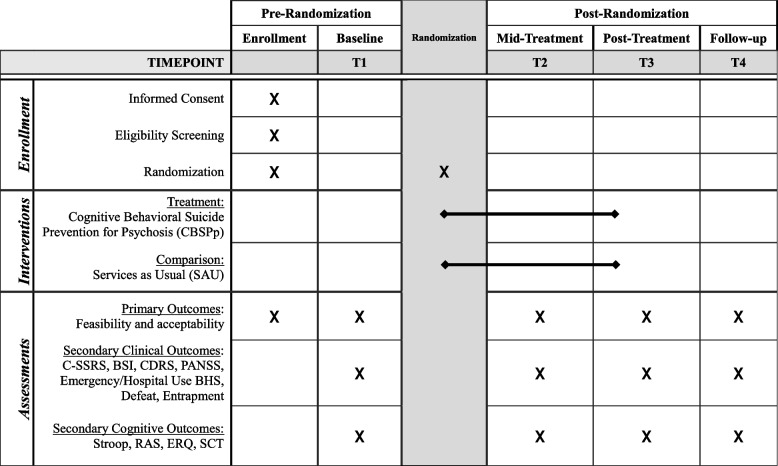
Client participant timeline and assessment schedule

Primary feasibility and acceptability outcomes include the following:

- The number of participants who give informed consent, are eligible to participate, enroll in the study, and complete study assessments will be tracked.
- The number and percentage of therapy sessions client participants in the treatment group attend will be tracked.
- Both client and provider participants will engage in in-depth qualitative interviews at T3 post-treatment for CBSPp acceptability explorations.

Secondary clinical and cognitive outcomes include the following:

- *Suicide ideation and/or attempt (SI/A): *Changes in suicide ideation and behavior will be assessed from baseline to T3 post-treatment using the Columbia-Suicide Severity Rating Scale (C-SSRS) [44] and Beck Scale for Suicide Ideation (BSSI) [54]. The following three questions are examples in the C-SSRS: “In the past month, have you had thoughts of killing yourself?,” “In the past month, had you started to work out or worked out the details of how to kill yourself?,” and “In the past month, have you made an attempt to end your life?” Ratings are coded as no (0) and yes (1). The presence and intensity of suicide thoughts are evaluated in the BSSI using 19 items with response categories ranging from 0 to 2 and a total score of 0 to 38, with higher scores indicating greater suicide ideation. Change in proportion of suicide attempt for the C-SSRS and in mean suicide ideation scores of the BSSI will be examined from baseline to T3 post-treatment and T4 follow-up.
- *Psychosis symptoms:* Changes in psychosis symptoms from baseline to T3 post-treatment and T4 follow-up will be assessed using the Positive and Negative Syndrome Scale (PANSS) [55], which has 30 items assessing positive, negative, and general psychopathology. The positive symptom subscale includes items related to organization, hallucinations, excitement, grandiosity, and suspiciousness/persecution. The negative symptom subscale includes items related to blunted affect, emotional withdrawal, poor rapport, passive/apathetic social withdrawal, difficulty in abstract thinking, lack of spontaneity and flow of conversation, and stereotyped thinking. Lastly, general psychopathology subscale includes items related to somatic concern, anxiety, guilt feelings, tension, mannerisms and posturing, depression, motor retardation, uncooperativeness, unusual thought content, disorientation, poor attention, lack of judgment and insight, disturbance of volition, poor impulse control, preoccupation, and active social avoidance. Rating anchors range from absent (1) to extreme (7) and items are summed to obtain a total score. Positive symptom subscale scores range from 7 to 49, negative symptoms from 7 to 49, and general symptoms from 16 to 112. Higher scores indicate greater presence and severity of symptoms and change in mean scores will be examined from baseline to T3 post-treatment and T4 follow-up.
- *Depressive symptoms:* Changes in depressive symptoms from baseline to T3 post-treatment and T4 follow-up treatment will be assessed using the Calgary Depression Rating Scale for Schizophrenia (CDRS) [56]. The following symptoms were measured within 1 week of assessment in 9 questions: depression, hopelessness, self-depreciation, guilty ideas of reference, pathological guilt, morning depression, early wakening, and observed depression. Ratings are coded as absent (0), mild (1), moderate (2), or severe (3), and items are summed to obtain a total score. Total scores range from 0 to 27 with higher scores indicating greater presence and severity of symptoms of depression and change in mean scores will be examined from baseline to T3 post-treatment and T4 follow-up.
- *Emergency and hospital service use:* Changes in emergency and hospital service use from baseline to T3 post-treatment and T4 follow-up will be assessed via service use data within electronic medical records. Data will indicate the prevalence of emergency and hospital service use and changes in the proportion of such use will be examined from baseline to T3 post-treatment and T4 follow-up.
- *Hopelessness:* Changes in hopelessness from baseline to T3 post-treatment and T4 follow-up will be assessed using the Beck Hopelessness Scale (BHS) [57]. The scale consists of 20 true-false items arrayed within three factors: Feelings about the future, loss of motivation, and future expectations. The total BHS score ranges from 0 to 20, with higher scores reflecting greater hopelessness, and change in mean scores will be examined from baseline to T3 post-treatment and T4 follow-up.
- *Defeat:* Changes in defeat from baseline to T3 post-treatment and T4 follow-up will be assessed using the 16-item Defeat Scale [58]. The scale assesses the perceptions of failed struggle and low social rank. Example items include, “I feel powerless” and “I feel there is no fight left in me.” Responses are rated on a 5-point Likert scale ranging from “Never” to “Always/all the time,” with higher scores indicating greater feelings of defeat. Change in mean scores will be examined from baseline to T3 post-treatment and T4 follow-up.
- *Entrapment:* Changes in entrapment from baseline to T3 post-treatment and T4 follow-up will be assessed using the 16-item Entrapment Scale [58]. The scale accesses the perception of being trapped and the desire to escape. Example items include, “I see no way out of my current situation” and “I feel powerless to change myself.” Responses are rated on a 5-point Likert scale ranging from “Not at all like me” to “Extremely like me,” with higher scores indicating greater feelings of entrapment. Change in mean scores will be examined from baseline to T3 post-treatment and T4 follow-up.
- *Information processing biases:* Changes in information processing biases from baseline to T3 post-treatment and T4 follow-up will be assessed using the modified Stroop task [59, 60]. This task instructs participants to name the font color (red or blue) of 48 words as quickly and accurately as possible. Words are either neutral (e.g., paper), positive (e.g., happy), negative (e.g., rejected), or suicide-related (e.g., dead). Participants press a red or blue key on a computer keyboard to indicate each word’s font color and reaction times are recorded using Empirisoft DirectRT v2004 software [61] or SuperLab 4.5.1 [62]. Mean response times, inference scores, and ratio scores are calculated for each of the word categories (neutral, positive, negative, and suicide), and changes will be examined from baseline to T3 post-treatment and T4 follow-up.
- *Appraisals:* Changes in appraisals from baseline to T3 post-treatment and T4 follow-up will be assessed using the Resilience Appraisals Scale (RAS) [63] and the Reappraisal Subscale of the Emotion Regulation Questionnaire (ERQ) [64]. The RAS includes 12 items and assesses for an individual’s ability to cope with emotions, solve problems, and gain social support to buffer one from suicide thoughts. Example items include, “I can handle my emotions” and “If I were to have problems, I have people I could turn to.” Responses are rated on a 5-point Likert scale ranging from “strongly disagree” to “strongly agree,” with higher scores indicating greater positive self-appraisals of one’s ability to cope with emotions, solve problems, and gain social support. The reappraisal subscale of the ERQ includes 6 items and assesses the extent to which participants use cognitive reappraisal as an emotion regulation strategy. Example items include, “When I want to feel more positive emotion, I change the way I’m thinking about the situation” and “When I’m faced with a stressful situation, I make myself think about it in a way that helps me stay calm.” Responses are rated on a 7-point Likert scale ranging from “strongly disagree” to “strongly agree,” with higher scores indicating greater use of cognitive reappraisal as an emotion regulation strategy. Change in mean scores will be examined from baseline to T3 post-treatment and T4 follow-up.
- *Schemas:* Changes in schemas from baseline to T3 post-treatment and T4 follow-up will be assessed using the Suicide Concept Sort Task (SCT) [45]. The task requires participants to sort a set of 10 cards from most closely related to least closely related to the construct of suicide. Words on each card include the following: sinful, beliefs, personality, hopeless, self-esteem, suffering, relieving pain, self-hate, psychosis, and death. The order of words illustrates a network of schema in relation to the construct of suicide. Change in the order of words will be examined from baseline to T3 post-treatment and T4 follow-up.

Exploratory outcomes include the following:

- *Psychological stress:* Changes in psychological stress from baseline to T3 post-treatment and T4 follow-up will be assessed using the Psychological Stress Index (PSI) [65]. The PSI is a 9-item measure of the susceptibility of individuals with psychosis to experience more negative affects in the face of daily life stressors, particularly those associated with interpersonal interactions, personal responsibilities, social expectations, and novel situations. Items are scored on a 5-point Likert scale ranging from never (0) to very often (4) with the total score calculated as a sum. Higher scores indicate greater psychological stress and change in mean scores will be examined from baseline to T3 post-treatment and T4 follow-up.
- *Coping:* Changes in coping from baseline to T3 post- treatment and T4 follow-up will be assessed using the Coping Inventory for Stressful Situations (CISS-21) [66] including 21 items to assess for three overarching coping strategies: Task-Oriented (T), Emotion-Oriented (E), and Avoidance. Items are scored on a 5-point Likert scale ranging from 1 (not at all) to 5 (very much), with subscale scores representing a sum. Higher scores indicate a greater use of a particular coping strategy, with avoidance and emotion-oriented coping being unhealthy coping approaches (higher scores are worse), and task-oriented coping being a healthy approach (higher scores are better). Change in mean scores will be examined from baseline to T3 post-treatment and T4 follow-up.
- *Stigma:* Changes in stigma from baseline to T3 post-treatment and T4 follow-up will be assessed using 3 items adapted from the Discrimination-Devaluation Scale (DDS) [67] to assess how participants perceive those who receive mental health treatment and how participants believe others perceive those who receive mental health treatment. Items are scored on a 6-point Likert scale ranging from 1 (strongly disagree) to 6 (strongly agree). Example items include, “Most people feel that receiving mental health treatment is a sign of personal failure” and “Most people think less of a person who has received mental health treatment.” Scores are summed with higher scores indicating stigma. Change in mean scores will be examined from baseline to T3 post-treatment and T4 follow-up.
- *Impulsivity:* Changes in impulsivity from baseline to T3 post-treatment and T4 follow-up will be assessed using 4 items from the Urgency Premeditated Perseverance Sensation Seeking Scale (UPPS) [68]. Items pertain to making regretful statements after rejection, finding it difficult to not act on feelings/emotions, making matters worse by acting without thinking when upset, and regretting impulsive actions. Response categories range from 0 (strongly disagree) to 3 (strongly agree), with total scores ranging from 0 to 12 and greater scores indicating greater impulsivity. Change in mean scores will be examined from baseline to T3 post-treatment and T4 follow-up.
- *Barriers to treatment:* Changes in treatment barriers from baseline to T3 post-treatment and T4 follow-up will be assessed using the following question about barriers to seeking services and/or engaging in services: “In the past 12 months, which of the following factors led you to receive fewer services (counseling, therapy, or medications) for your mental or behavioral health?” This question was followed by a list of 24 barrier types used in prior studies of college students [69] in which various attitudes, beliefs, and experiences represent help-seeking barriers that lead to no service use. Consistent with prior research on help-seeking barriers using this assessment tool [70–72], barrier types were categorized into the following: time (lack of time), fear of stigma (worries about loss of privacy and stigma), financial concerns (limited or lack of financial resources), questioning (doubts about usefulness of therapy and need for help), logistics (practical issues related to treatment access), and cultural sensitivity/understanding (sensitivity to issues affecting gender, sexual, or racial/ethnic identities). Barrier scores range from 0 to 24, with higher scores indicating greater barriers experienced. Change in the proportion of barriers will be examined from baseline to T3 post-treatment and T4 follow-up.
- *Provider evidence-based attitudes:* Changes in provider participant attitudes towards evidence-based practice (EBP) from baseline to T3 post-treatment and T4 follow-up will be assessed using the Evidence-Based Practice Attitudes Scale (EBPAS) [73]. The scale includes 15 items to examine provider willingness and openness to adopt EBPs, along with perceived importance of using evidence-based interventions in practice. Items were scored on a 5-point Likert scale representing the extent to which a provider agrees with each statement, ranging from not at all (0) to a very great extent (4). The total and subscale mean scores range from 1 to 5 with higher scores indicating greater requirement, appeal, and openness to using EBPs in practice. Change in the order of words will be examined from baseline to T3 post-treatment and T4 follow-up.

## Data analysis plan

Quantitative assessment data will be examined descriptively (mean, standard deviation, percentage) to describe the demographic and clinical characteristics of the sample and examine the size of any chance imbalances between the two groups.

### Primary feasibility and acceptability data analysis

The feasibility parameters and criteria for success shown in Table 2 will be estimated to inform the design of a subsequent larger trial to evaluate CBSPp. Feasibility parameters involve the recruitment rate, CBSPp treatment engagement and adherence, and rates of completion and retention in the study and treatment for participants randomly selected to deliver or receive CBSPp. Study participants and CBSPp group dropouts who complete T1 but not T2–T4 will be compared to those who complete the study. Participant characteristics will be evaluated as predictors of dropout using regression and survival analyses will be conducted to examine length of participation in treatment. If all or some fidelity parameter criteria for success in Table 2 are not met, investigators will examine contributing factors to feasibility findings and pursue modifications as needed to the treatment and its delivery prior to a subsequent larger trial.

**Table 2:** Feasibility parameters

Parameter	Data collection methods	Criteria for success
Recruitment rate	Client participants: The number of clients who give informed consent to be screened for study participation, are eligible to participate in the study, and enroll in the study, versus those who decline (reasons will be recorded) Provider participants: The number of providers who give informed consent to participate in the study and enroll in the study, versus those who decline (reasons will be recorded)	Between 2 and 3 eligible and enrolled client participants recruited each month over a 2-year period, totaling 60 client participants by the end of study Between 6 and 10 eligible, enrolled, and CBSPp-trained provider participants recruited over a 2-year period
Treatment engagement and adherence	Client participants: The number of clients in the CBSPp group who begin treatment (attend session 1), attend each of the 10 individual treatment sessions, and the mean and median number of all treatment sessions attended Provider participants: The number of providers who complete the CBSPp training, pass the knowledge examination and fidelity role-plays prior to delivery, pass fidelity evaluations of 20% randomly selected sessions in the study, attend twice monthly supervision, begin to deliver treatment (deliver session 1), deliver each of the 10 individual treatment sessions, and the mean and median number of all treatment sessions delivered	≥ 80% of client participants recruited commencing treatment (the CBSPp group) ≥ 80% of provider participants recruited commencing treatment (the CBSPp group)
Completion and retention rates	The number of clients and providers who complete the 10 individual treatment sessions, study assessments at four timepoints (T1, T2, T3, and T4), versus those who do not complete treatment and/or study assessments (reasons will be recorded)	≥ 75% of participants completing the 10 individual treatment sessions and study assessments (providing data) at T3 and T4

Descriptive statistics will be reported for quantitative survey questions and in-depth qualitative interview questions regarding CBSPp experience and acceptability from the perspectives of provider and client participants. Acceptability topics will focus on perceived expectations, benefits, motivations, and barriers in relation to study participation and engagement in CBSPp, if in the treatment group.

### Secondary clinical and cognitive outcome data analysis

All analyses for outcome data will use the entire intent-to-treat sample. Linear mixed models (LMM) will be used for continuous outcomes and generalized linear mixed models (GLMM) for dichotomous outcomes to assess whether CBSPp is associated with improvements in clinical (suicide ideation and/or attempt (SI/A), symptoms of psychosis, depression, and emergency/hospital service, hopelessness, defeat, and entrapment) and cognitive (information processing biases, appraisals, and schemas) outcomes from T1 (baseline) to T3 (post-treatment). Parameter estimates for the interaction between group membership and time will determine if the CBSPp treatment group has significantly different changes over time than the comparison SAU group. Baseline values of the outcome variables and other baseline covariates found to be related to the outcome variable or missingness will be considered covariates in the mixed models.

In relation to treatment dropout and per the intent-to-treat principle, we will aim to motivate participants to adhere to the scheduled measurements (T1–T4) should they stop treatment in the CBSPp group. Multiple imputation methods [74] will be used to handle missing data after T1. In addition, sensitivity analyses using selection modelling and pattern mixture modelling [75] will be performed to examine the potential influence of missingness on findings.

### Exploratory data analysis

We will explore potential mechanisms of CBSPp effectiveness (i.e., decreased SI/A, psychosis, depression, and service use) by analyzing potential mediators (i.e., hopelessness, defeat, entrapment, social support, coping, and suicide-related information processing biases, appraisals, and schemas) and moderators (e.g., illness duration, history of suicidal ideation and behavior, psychosis symptom profile). To test mediation, we will use path analysis implemented in Mplus and test indirect effects. To test moderation, we will include interaction terms between the possible moderator and group membership in the mixed model.

### Qualitative data analysis

Qualitative in-depth interview data about treatment experience from provider and client participants at T3 will be transcribed and coded using Dedoose. Transcripts will be independently coded by two research staff in preparation for codebook development using an open coding technique to generate themes across qualitative questions [76]. Grounded theory methods were utilized for analysis [77]. The PI will meet with research staff to discuss emerging themes after a first round of coding and agreed upon a final codebook. Research staff will complete a second round of coding using the final codebook, and the PI will resolve potential disagreements to achieve inter-coder consistency. Themes will ultimately be organized into a final framework, and the following strategies for rigor [78] will be included in the study: (1) triangulation, specifically analytic triangulation (more than one coder), (2) audit trail, and (3) member checking with stakeholder participants (i.e., in provider, peer, and client meetings).

## Discussion

This paper describes a protocol for the first pilot acceptability and feasibility randomized clinical trial of CBSPp modified for a CMH setting in the USA aiming to investigate the acceptability, feasibility, and preliminary effectiveness. The primary objective of this study is to examine the feasibility and acceptability of modified CBSPp. The secondary objective of this study is to preliminarily evaluate whether modified CBSPp is associated with reductions in clinical (suicide ideation and/or attempt (SI/A), symptoms of psychosis, depression, and emergency/hospital service, hopelessness, defeat, and entrapment) and cognitive (information processing biases, appraisals, and schemas) outcomes. If CBSPp is feasible, acceptable, and demonstrates preliminary effectiveness, it will result in an innovative suicide prevention intervention for adults with psychosis to be implemented and further examined in CMH settings across the US.

This protocol has several important strengths. First, it will examine the feasibility, acceptability, and preliminary effectiveness of an innovative cognitive-behavioral treatment with specific tailoring for psychosis symptoms to reduce and prevent SI/A. Second, participants with psychosis symptoms and SI/A are included in the study, an intersection of mental health experiences that are often not well-represented in clinical trials due to exclusion criteria [33]. Third, multidimensional standardized assessments will be used to evaluate change in various clinical and cognitive constructs with blinding for raters of diagnostic and clinical interviews. Fourth, there will be longitudinal measurement of four timepoints including pre-treatment, during-treatment, post-treatment, and follow-up. Fifth, an innovative and rigorous hybrid approach to training and establishing fidelity for providers will be tested.

Despite the protocol’s significance and strength, there are limitations to note. First, the sample will be recruited from one CMH setting in a mid-western region of the US. Therefore, the sample may differ from other mental health settings or geographical areas and will not be representative of all clients with psychosis and SI/A. Second, participants will be current clients of the CMH setting with a desire to receive this treatment and be involved in research. Therefore, the sample may differ from individuals not engaged with services or not wanting to receive a suicide prevention treatment. Our future research will explore ways to increase CBSPp appeal and interest among clients who are not treatment seeking or engaged in services. Third, this study has insufficient power to determine the effectiveness of CBSPp on all outcomes of interest given the sample size. Given we expect approximately 60 clients total, with 30 randomized to each study group, our goal is to establish preliminary effectiveness with this protocol as a step towards future studies including larger samples and multiple clinical sites.

Suicide is among the leading causes of death in this population with a paucity of psychosis-specific evidence-based interventions to reduce SI/A; thus, it is critical to develop and test evidence-based services to support this public health need and reduce premature suicide death in this at-risk population. CBSPp is one of few cognitive-behavioral interventions designed for adults with psychosis symptoms and SI/A and was modified by our team for a CMH setting in the US. By establishing the feasibility, acceptability, and exploring preliminary effectiveness of modified CBSPp, this pilot trial will lead to further testing and dissemination of a promising approach to suicide prevention with tailoring for individuals with psychosis symptoms.

## Data Availability

All data will be submitted to the National Institute of Mental Health (NIMH) Data Archive (NDA). All investigators will have access to the finalized dataset.

## Abbreviations

AE: Adverse events
ANOVA: Analysis of variance
BHS: Beck Hopelessness Scale
CBSPp: Cognitive-Behavioral Suicide Prevention for Psychosis
CDRS: Calgary Depression Rating Scale
CISS: Coping Inventory for Stressful Situations
CMH: Community Mental Health
CREST: Clinical Research Education Support and Training
C-SSRS: Columbia-Suicide Severity Rating Scale
Co-Is: Co-investigators
DDS: Discrimination-Devaluation Scale
DMC: Data Monitoring Committee
DSM-5: Diagnostic and Statistical Manual of Mental Disorders, 5th Edition
DSMB: Data Safety and Monitoring Board
EBP: Evidence-based practice
EBPAS: Evidence-based Practice Attitudes Scale
ERQ: Emotion Regulation Questionnaire
GLMM: Generalized linear mixed models
ICF: Informed consent form
IRB: Institutional Review Board
LMM: Linear mixed models
NDA: NIMH Data Archive
NIH: National Institutes of Health
NIMH: National Institute of Mental Health
MINI: Mini International Neuropsychiatric Interview
PANSS: Positive and Negative Syndrome Scale
PI: Principal investigator of the grant (Dr. Lindsay Bornheimer)
PSI: Psychological Stress Index
RAS: Resilience Appraisals Scale
SAU: Services as usual
SAS: Statistical analysis system
SAE: Serious adverse events
SI/A: Suicide ideation and attempt
SMI: Serious mental illness
SSD: Schizophrenia spectrum disorders (*no longer SSPD [schizophrenia spectrum and other psychotic disorders])*
SCT: Suicide concept sort task
T1 / T2 / T3 / T4: Timepoint 1 / 2 / 3 / 4
UPPS: Urgency Premeditation Perseverance Sensation Seeking Scale
WRAT: Wide Range Achievement Test
